# Supplementary material for: CEO’s functional experience and firm performance based on text mining
Source: PLoS One. 2023 Mar 17;18(3):e0281866. doi: 10.1371/journal.pone.0281866 (PMC10022806; doi:10.1371/journal.pone.0281866)
Supplement: Attachment — Submitted filename: Review_Plos One.pdf [file pone.0281866.s001.pdf]

Manuscript number: PONE-D-22-03262

Title: CEO's Functional Experience and Firm Performance Based on Text Mining

I would like to thank you for giving me the opportunity to review the article. In this paper, authors investigate the impact of CEO's functional experience on firm performance using a text mining approach. They find that CEO's breadth and depth in terms of experience and background have nonlinear impacts on firm performance. I found the paper interesting but have several major concerns particularly about the motivation and methodology which were stated below.

1) The introduction section of the paper does not clearly address the motivation and the contribution of the paper. As authors state in the introduction, there are several studies in the literature which investigate the role of CEO background or experience on firm performance or other financial policies. However, it is quite not clear in the introduction that in which ways this paper differentiates from the other papers in the literature. Authors state that "the purpose of the paper was to increase the interest of researchers in continuing to study CEO's functional experience characteristics and their effects on firm performance". I am not quite sure if this statement clearly identifies the objectives of research. What are the specific aims or research questions of the study and how they contribute to the literature? Authors also claim that the main contribution of the study is using a "cutting-edge information technology methods". I think authors should also extend this part by a few sentences to make the contribution of the paper clearer in the introduction. Overall, authors could revise the introduction section to make it more informative for the readers.

2) Authors may extend the literature review part by including more studies which directly investigate the role of CEO experience in determining firm performance. For example, Hamori and Koyuncu (2015) investigate the impact of prior CEO experience on firm performance. In addition, Li and Patel (2019) investigate the impact of CEO generalist experience and firm performance. Also, Georgakakis and Ruigrok (2017) provide evidence on the relationship CEO characteristics and background, and organizational performance. I couldn't find any discussion about these studies. It would be useful to extend the literature by including more studies.

   References

   Hamori, M., & Koyuncu, B. (2015). Experience matters? The impact of prior CEO experience on firm performance. *Human Resource Management, 54*(1), 23-44.

   Li, M., & Patel, P. C. (2019). Jack of all, master of all? CEO generalist experience and firm performance. *The Leadership Quarterly, 30*(3), 320-334.

   Georgakakis, D., & Ruigrok, W. (2017). CEO succession origin and firm performance: A multilevel study. *Journal of Management Studies, 54*(1), 58-87.

3) Regarding Section 3.1, authors claim that CEO's functional experience increases the "instability" and this instability negatively affects the firm performance. I am not quite

confident about this argument. What does “instability” refer to? Is it instability in deciding corporate policies or in financial performance? Also it is not clear why CEO experience increases instability.

4) Related to my previous comment, authors also state that “This instability affects the company’s performance and has a greater risk-averse ability”. This argument is also not clear. I am not quite sure about what “greater risk-averse ability” means? I think authors make their arguments more clear and direct when setting up the hypotheses. It is also essential to support these arguments with the literature.

5) Authors use ROA and ROE as the performance measures. It is useful to consider Tobin’s Q as the market-based performance measure in addition to the ROA and ROE which are accounting based measures.

6) I am not comfortable with the data definitions. Authors use (total profit + interest expense) scaled by Average total assets. Not sure why authors add interest expense to the total profit. Also, ROE definition is not correct. Authors scale net profit by net assets to calculate ROE. It should be scaled by Equity. I suggest authors to follow the papers published in reputable journals when defining the variables.

7) Authors employ PSM to address selection bias and the results are reported in Table 3 and Column 5. However, it is not clear how PSM has been employed. Considering the number of observations for each Column in Table 3, authors manage to match each firm in the treated group with another firm in the control sample because there is no decrease in the number of observations which is quite uncommon in PSM analysis. Then, what is the difference between Column 3 and Column 5 in Table 3?

8) Section 5.2.2 is also confusing. Is there any definition for KMO? Authors state that first principal component explains 76.48% of the total variables. How did authors end up with this number? Are PEF (in the text) and PET (in Table 3) the same? Authors should revise this part to avoid any confusion.

9) Authors should check the reference list for any mistakes. For example, Reference 48 has been published in “Journal of Finance and Bank Management” not in the “Journal of Finance”. It would be useful to check references to avoid any typos and mistakes in the Journal names.

10) I also recommend authors to avoid citing papers in unknown journals.

11) There are several typos in the text. It would be useful to review/proofread the text to avoid any typos and grammatical errors.
