# Supplementary material for: CEO’s functional experience and firm performance based on text mining
Source: PLoS One. 2023 Mar 17;18(3):e0281866. doi: 10.1371/journal.pone.0281866 (PMC10022806; doi:10.1371/journal.pone.0281866)
Supplement: Attachment — Submitted filename: Plos one comments.pdf [file pone.0281866.s003.pdf]

Manuscript number: PONE-D-22-03262_R1
Title: CEO’s Functional Experience and Firm Performance Based on Text Mining

Authors made some improvements in the paper based on the comments provided in the previous round. However, I still have some concerns regarding particularly data and methodology section which were stated below.

1) Introduction section now clearly states the motivation and the contributions of the paper.

2) It would be useful to add references for calculating func_rich and level_score. For example, authors use a weighted score of 6:3:1 for senior, middle, and junior levels. Is there any reason for using a weight of 6 for senior levels and 3 for middle levels? As they are the main variables of interest, authors should be more precise and clearer when explaining these variables.

3) ROA explanation is still not correct. It should be “the ratio of net income and total assets” not “total income”.

4) Degree variable is a categorical variable, but it is included in the regression as a continuous variable (as I understand from the results table). Authors should check which variables are continuous and categorical.

5) It is good to include Tobin’s Q variable as another proxy for the firm performance. However, there is no discussion about it. How is it calculated? Is it only an alternative to ROA and ROE (for robustness purposes) or another performance proxy such as PEF? It would be useful to provide more discussion/explanations about it.

6) Authors claim that they are using firm fixed effects regression in Section 4.3 based on Hausman test, but Table 3 suggests “Industry fixed effects”. I am not quite sure if authors use firm or industry fixed effects.

7) I am still not convinced about the endogeneity test. Authors provide some graphs (descriptive) about the relationship between CEO and top management teams changes, but it is not clear how it addresses endogeneity. What should readers understand from these graphs? Are there any other sources of endogeneity such as omitted variables, selection bias etc.? After explaining the Figure 2, authors state that “the model results still support Hypothesis 1 and 2”. I am not quite sure which model authors refer to and how it supports Hypothesis 1 and 2.
