# Supplementary material for: CEO’s functional experience and firm performance based on text mining
Source: PLoS One. 2023 Mar 17;18(3):e0281866. doi: 10.1371/journal.pone.0281866 (PMC10022806; doi:10.1371/journal.pone.0281866)
Supplement: Attachment — Submitted filename: Response to Reviewers.docx [file pone.0281866.s002.docx]

Dear Editor,

Thank you for your valuable comments and suggestions on the structure of our manuscript. We have modified the manuscript accordingly, and the main corrections in the paper and the responses to the referees' comments are as follows:

**Reviewer #1**

Q1: The introduction section of the paper does not clearly address the motivation and the contribution of the paper. As authors state in the introduction, there are several studies in the literature which investigate the role of CEO background or experience on firm performance or other financial policies. However, it is quite not clear in the introduction that in which ways this paper differentiates from the other papers in the literature. Authors state that “the purpose of the paper was to increase the interest of researchers in continuing to study CEO’s functional experience characteristics and their effects on firm performance”. I am not quite sure if this statement clearly identifies the objectives of research. What are the specific aims or research questions of the study and how they contribute to the literature? Authors also claim that the main contribution of the study is using a “cutting-edge information technology methods”. I think authors should also extend this part by a few sentences to make the contribution of the paper clearer in the introduction. Overall, authors could revise the introduction section to make it more informative for the readers.

Re: We apologize for the poorly considered experiment. Accordingly, we added the motivation of the paper.

The relationship between CEO function experience and firm performance seems to be a black box problem. Thus, how to further analyzing this problem has motivated the researchers to carry out this study. Many studies support the idea that CEO experience affects firm performance. However, there are still some shortcomings in the existing research. (1) The breadth-based research does not consider the diversity and duplication of functions, and the rough count of the number of positions is not enough to reveal the breadth of the CEO's service experience. (2) The depth-based research does not consider the tenure of each stage of employment, and these studies do not have a hierarchy of positions. (3) Current data analysis and processing methods are not always scalable, especially manual processing methods cannot automate research on a wide range of listed companies. Hence, these factors motivated the researchers to conduct this study.

Q2: Authors can extend the literature review part by including more studies which directly investigate the role of CEO experience in determining firm performance. For example, Hamori and Koyuncu (2015) investigate the impact of prior CEO experience on firm performance. In addition, Li and Patel (2019) investigate the impact of CEO generalist experience and firm performance. Also, Georgakakis and Ruigrok (2017) provide evidence on the relationship CEO characteristics and background, and organizational performance. I couldn’t find any discussion about these studies. It would be useful to extend the literature by including more studies..

Re: Thank you very much for your suggestion. We have carefully read the literature you provided and added it to our paper. At the same time, we add some recent literature to strengthen the study further.

Q 3: Regarding Section 3.1, authors claim that CEO’s functional experience increases the “instability” and this instability negatively affects the firm performance. I am not quite confident about this argument. What does “instability” refer to? Is it instability in deciding corporate policies or in financial performance? Also it is not clear why CEO experience increases instability.

Re: We are mainly referring to the instability of the environment and decision-making. Recent studies have found that CEO's experience hinders subsequent firm performance (44, 45). The rationale is that prior experience is too heavily laden with the specific environments in which it was gained and therefore is not as beneficial to the new firms as the CEOs believe it will be. In short, available evidence suggests that past experience is a detriment to success in a subsequent job, mainly because experienced CEOs have to give up firm-specific skills that are not useful to a new firm (45). It is exacerbated by the tendency of CEOs to become less adaptable with increasing experience (40, 46, 47). Therefore, as the CEO's functional experience increases, they face more and more instability in the environment and decision-making, which is not conducive to improving the firm performance (48).

Q4: Related to my previous comment, authors also state that “This instability affects the company’s performance and has a greater risk-averse ability”. This argument is also not clear. I am not quite sure about what “greater risk-averse ability” means? I think authors make their arguments more clear and direct when setting up the hypotheses. It is also essential to support these arguments with the literature.

Re: The upper echelons theory believes that the individual's experience characteristics also affect risk preference (1). When a listed company hires a CEO, their experience is usually considered. In reality, more and more listed companies hire experienced CEOs because they can provide more professional policy advice for the company's development. On the one hand, experienced CEOs can apply their expertise to practical decisions, helping to make sound decisions. On the other hand, reliable and prudent practice requirements will also prompt them to form corresponding characters and habits with a lower risk appetite (49).

Q5: Authors use ROA and ROE as the performance measures. It is useful to consider Tobin’s Q as the market-based performance measure in addition to the ROA and ROE which are accounting based measures.

Re: Tobin's Q: Ratio of the market value of the firm to total assets. The regression results of the Tobinq indicator are added to the robustness test of this paper. The specific results are shown in Table 3.

Q6: I am not comfortable with the data definitions. Authors use (total profit + interest expense) scaled by Average total assets. Not sure why authors add interest expense to the total profit. Also, ROE definition is not correct. Authors scale net profit by net assets to calculate ROE. It should be scaled by Equity. I suggest authors to follow the papers published in reputable journals when defining the variables.

Re: Thank you for your carefully and patiently reviewing the manuscript. We are sincerely sorry for our manuscript's incorrect definition of those variables. We have already corrected the description according to your valuable suggestion in the revised manuscript.

ROA: Return on Assets i.e. the ratio of total income and total assets.

ROE: Return on Equity i.e. ratio of net income to shareholder's equity.

Q7: Authors employ PSM to address selection bias and the results are reported in Table 3 and Column 5. However, it is not clear how PSM has been employed. Considering the number of observations for each Column in Table 3, authors manage to match each firm in the treated group with another firm in the control sample because there is no decrease in the number of observations which is quite uncommon in PSM analysis. Then, what is the difference between Column 3 and Column 5 in Table 3?

Re: Our paper uses the sample median of func_rich as the benchmark to divide the sample into experimental and control groups for the PSM test. After careful consideration, it is found that there is indeed a problem with the PSM made in this article. To further address this issue, we propose a robustness check that we believe is better.

A potential problem with our findings is that listed companies have other executives besides the CEO. According to our statistics, the top management team changes almost every year. If the top management team is highly fluid, it is difficult to measure whether the CEO or other executives bring about an improvement in firm performance.

Therefore, this paper analysis the stability of the CEO and top management team each year. Figure 1 shows the CEO and top management team changes over the statistical period as a group of company representatives. The blue dotted line indicates whether the CEO has changed in the current year, 1 if the CEO changes, and 0 if there is no change. The solid red line represents the rate of change in the top management team.

**Figure 1 The change rate of the CEO and Top Management Team**

As can be seen from the figure, most listed companies have changed their CEOs during the statistical period. The changes in the top management team are different. Some companies have undergone substantial changes in the top management team during the statistical period, while the changes were relatively small in others. We further makes a descriptive statistic of the change rate of the top management team. The rate of change of the top management team is distributed between [0, 1]. The average rate was 13%. Therefore, this paper believes that if the current year's change rate of the top management team is less than 13%, it is relatively stable. According to this standard, this paper filtered the data of CEO changes and a relatively stable top management team for model testing. The part of sample are shown in Figure 2.

**Figure 2 The change rate of the CEO and Top Management Team in the filtered samples**

The filtered samples are smaller but cleanest. In these samples, it is guaranteed that the CEO changes, but the top management team remains relatively stable. This method can better describe the relationship between CEOs' functional experience and company performance. The model empirical analysis results for this sample are in column 7 in Table 3. The model results still support Hypotheses 1 and 2.

Q8: Section 5.2.2 is also confusing. Is there any definition for KMO? Authors state that first principal component explains 76.48% of the total variables. How did authors end up with this number? Are PEF (in the text) and PET (in Table 3) the same? Authors should revise this part to avoid any confusion.

Re: PEF (in the text) and PET (in Table 3) the same. We have unified. The KMO (Kaiser-Meyer-Olkin) test statistic is an indicator used to compare simple and partial correlation coefficients between variables. It is mainly used in factor analysis of multivariate statistics. We are very sorry for confusing the two concepts, this paper used PCA (Principal Component Analysis) to analyze the indicators.

PCA is to try to recombine the original many related indicators (such as P indicators) into a new set of comprehensive indicators independent of each other to replace the original ones. The results of the principal component analysis are shown in Table 4. Among them, (Component) represents the name of the principal component extracted by the system. It can be found that a total of 5 principal components have been extracted. The Eigenvalue column represents the eigenvalue of the principal component extracted by the system. The size of the eigenvalue means the explanatory power of the principal component. The larger the eigenvalue, the stronger the explanatory power. The Proportion column represents the variance contribution rate of the principal component extracted by the system, and the variance contribution rate is also the explanatory power of the principal component.

Table 4 Regression results

| Principal components/correlation | | | | |
| --- | --- | --- | --- | --- |
| **Component** | **Eigenvalue** | **Difference** | **Proportion** | **Cumulative** |
| Comp1 | 2.4820 | 2.0799 | 0.8273 | 0.8273 |
| Comp2 | 0.4021 | 0.2862 | 0.1340 | 0.9614 |
| Comp3 | 0.1158 | 0.0001 | 0.0386 | 1 |
| Principal components (eigenvectors) | | | | |
| **Variable** | **Comp1** | **Comp2** | **Comp3** | **Unexplained** |
| ROA | 0.5861 | -0.4998 | 0.6378 | 0 |
| ROE | 0.6049 | -0.2539 | -0.7548 | 0 |
| TobinQ | 0.5392 | 0.8281 | 0.1535 | 0 |

Ultimately, the characteristic root of the first principal component is 2.4820, which is greater than 1. The first principal component explains 82.73% of the total variables. The expression of the composite indicator of firm performance (PEF) obtained by expressing the first principal component as a linear combination of the individual variables is:

$PEF=0.5861ROA+0.6049ROE+0.5392TobinQ$	(5)

The empirical test was conducted with TobinQ and PEF as the dependent variable. As shown in column (5) and (6) in Table 3, the regression results still support hypothesis one and hypothesis two.

Q9: Authors should check the reference list for any mistakes. For example, Reference 48 has been published in “Journal of Finance and Bank Management” not in the “Journal of Finance”. It would be useful to check references to avoid any typos and mistakes in the Journal names.

Re: In the last version, Reference 48 is: *Azeez A A. Corporate governance and firm performance: evidence from Sri Lanka[J]. Journal of Finance, 2015, 3(1): 180-189.* The reference is indeed from “Journal of Finance”. To avoid ambiguity, we removed this reference.

Q10: I also recommend authors to avoid citing papers in unknown journals.

Re: Thanks for your suggestion, we have taken your advice and updated the references.

Q11: There are several typos in the text. It would be useful to review/proofread the text to avoid any typos and grammatical errors.

Re: We have a try to correct the spelling errors and paragraphs to make them more readable.

**Reviewer #2:**

Q1: I am happy to review this research work. It is unique in nature and offers novel findings, particularly in the Chinese context. Overall it's well written, while I have only one suggestion which makes further interesting finds of this work, that is the female CEO. The author(s) explained well the variable measurement along with gender classification (male 1, female 0). I suggest adding a new hypothesis that is exactly the same (H1, H2) which deals with the female CEO breath and depth, Author(s) made a huge effort to sort the data over 25000 observations covered the period 2006-2020 which is good. This is just my suggestion, if the author(s) faced serious difficulty to grab/splitting the same data into a female context then the author(s) may continue as it is.

Re: We do appreciate your comments. With the rapid development of the social economy, more and more female managers have entered top management and achieved excellent results. From the perspective of female CEOs, studying their impact on firm performance is not only a realistic response to contemporary corporate research but also reflects the demand trend of contemporary research. With the improvement of female status, especially the rapid increase in female CEOs in modern firms, it is of great theoretical and practical significance to study the impact of female CEOs' functional experiences on firm performance.

In China, however, females often work harder to achieve the same status as males. Females' ability to work is often underestimated, and not many of them can become CEOs of public companies. Through the statistical analysis of the data in this paper, in the 15-year statistical period, female CEOs only accounted for 6.4%, and the ratio of males and females was seriously unbalanced.

We split the data into male and female CEOs, then analyzed the breadth and depth of female and male CEOs' functional experiences and found some interesting results. First, Figures 3 shows the descriptive statistics of the two subsamples. Figures 4 and 5 are the position promotion charts for female and male CEOs. We found those female CEOs had a wider breadth of functional experience than males, with an average of 0.9054 but 0.5159 for males. However, in terms of depth of experience (level_score and avg_emp), female CEOs generally have a lower depth of experience.

**Figure 3** Descriptive statistics of the two subsamples

**Figure 4 Evolution of CEO's position promotion chart of female CEOs**

**Figure 5 Evolution of CEO's position promotion chart of male CEOs**

The above is only a preliminary exploration, the comments are of great value to this paper, and we also found some highlights of the exploration. However, we believe that analyzing female and male CEOs still faces many problems and needs complete data and theoretical support. Thanks for your suggestion; this is what we need to work on in the future. We are also working on this; you will see research in this area in our future work.

Q2: Indeed, it is new work, with novel findings while the author(s) may add some recent literature to further strengthen the study. Currently, there is no study referring to 2022 and very few studies refer to 2021.

Re: Thank you very much for your suggestion. We have added some recent literature to strengthen our study.

Q 3: The author (s) used an industry-year fixed effect in the model which seems that the author used a fixed effect model rather than random or others. There is no discussion about the diagnostics test, I believe it must be checked during the model/analysis selection but if it is mentioned in the study it also strengthens the analysis/results justifications.

Re: This is a constructive suggestion to improve the paper's publication potential. According to the characteristics of panel data, we test the validity of the fixed-effects model and the random-effects model on the validity of the regression model. Usually, the Hausman test is used to judge the applicability of the two models (see Figure 6). The p-value of the Hausman test result is 0.0000, so the null hypothesis is strongly rejected, and a fixed-effects model should be used instead of a random-effects model.

**Figure 6 The result of Hausman test**

Thank you for your patiently reviewing the manuscript. We are sincerely sorry for our carelessness. We have already corrected the description according to your valuable suggestion in the revised manuscript. We hope that you will be satisfied with the revision. Subsequently, we look forward that the revised manuscript being adaptable for the demands for open publication.

I want to re-submit this manuscript and hope it is acceptable for publication in the journal. If there are any questions about our paper, please do not hesitate to let us know. Thank you very much for your attention to our paper.

Best regards,

Xiaohong Huang

School of Logistics and Management Engineering

Yunnan University of Finance and Economics

Kunming, 650221, China

E-mail: huangxiaohong@163.sufe.edu.cn
