# Supplementary material for: CEO’s functional experience and firm performance based on text mining
Source: PLoS One. 2023 Mar 17;18(3):e0281866. doi: 10.1371/journal.pone.0281866 (PMC10022806; doi:10.1371/journal.pone.0281866)
Supplement: Attachment — Submitted filename: Response to Reviewers.docx [file pone.0281866.s004.docx]

Dear Editor,

Thank you for your valuable comments and suggestions on the structure of our manuscript. We have modified the manuscript accordingly.

Q1: Please note that you don't have to worry about the first comment related to the contribution but if you can, try to clarify the motivation (I encourage you doing so) and a bit of contribution for future readers (but not compulsory).

Re: Thank you very much for your suggestion. We have carefully read some relation literature and clarify the motivation and contribution of our paper as follow.

**Motivation** : From a simple statistical view, some representative companies, such as Tencent and Alibaba, have achieved excellent firm performance, but the experience of their CEOs is very different. Moreover, there are also other CEOs whose experience falls somewhere in between. From the CEOs' view, we were interested in the differences in their experience. At the same time, it is also worth paying attention to the direction of the firm performance results of these differences. The question arises: What characteristics of a CEO's experience are more conducive to improved firm performance? Examining this question may enable us to better understand the motivation for CEO selection and facilitate a reasonable and targeted CEO evaluation.

**Contributions**: Our study makes three contributions to the literature. First, compared with the subjective judgment of manual collation and questionnaires, this paper processed the data using cutting-edge information technology methods such as named entity recognition. We collected CEOs' resume data for 15 consecutive years from 2006 to 2020 to obtain comprehensive experience details. The analysis results are scientific and objective, and the technical approach can be extended to other types of executives. Second, compared with studies that mainly focus on the job category and tenure of executives' experience, this article introduced the breadth and depth dimensions of CEOs' functional experience enriching the study of the upper echelons theory. Finally, a few studies have provided partial evidence of the relationship between a CEO's experience and firm performance. In addition to the direct effect of the CEO's experience on firm performance, this paper further emphasized the nonlinear relationship between the two dimensions. The findings of this paper may serve as direct evidence for related studies to expand the literature on CEOs' experiences.

Q2: To avoid further delay, at this stage, I would encourage you using professional proof reading service to improve the writing style and correct grammatical and spelling errors.

Re: Thank you for your patiently reviewing the manuscript. We are sincerely sorry for our carelessness. We have already corrected the description according to your valuable suggestion in the revised manuscript.

**Reviewer**

The main corrections in the paper and the responses to the reviewer's comments are as follows:

Q1: Introduction section now clearly states the motivation and the contributions of the paper.

Re: Thank you very much for your affirmation. At the same time, combined with the comments of the editor, we further clarified the motivation and the contributions.

Q2: It would be useful to add references for calculating func_rich and level_score. For example, authors use a weighted score of 6:3:1 for senior, middle, and junior levels. Is there any reason for using a weight of 6 for senior levels and 3 for middle levels? As they are the main variables of interest, authors should be more precise and clearer when explaining these variables .

Re: We apologize for the poorly considered explanation. Accordingly, we added the explanation of the weighted score. On August 31, 2006, the Ministry of Human Resources and Social Security of the People's Republic of China issued the Implementation Opinions of the Tentative Measures for the Management of Post Establishment in Public Institutions. According to Article 13 of the setting of professional and technical post levels states: the structural proportion of senior, intermediate, and junior posts shall be controlled according to the regional economic and social development level and industrial characteristics, as well as the function, specification, subordination and professional and technical level of the public institution. The overall national control target is 1:3:6 for the proportion of senior, intermediate, and junior posts.

Combined with the structural ratio of senior, intermediate, and junior positions, this paper gave a weighted score of 6:3:1 to senior, intermediate, and junior levels.

We have added references and links as follows:

*[56]MoP. Administrative Measures for Post Setting in Public Institutions. Administrative Measures for Post Setting in Public Institutions. 2006(70). http://www.mohrss.gov.cn/SYrlzyhshbzb/rencairenshi/zcwj/shiyedanweirenshiguanli_1/202002/t20200210_358464.html*

Q3: ROA explanation is still not correct. It should be “the ratio of net income and total assets” not “total income” .

Re: Thank you for your patiently reviewing. We are sincerely sorry for our carelessness. We have already corrected the explanation of ROA according to your valuable suggestion in the revised manuscript.

Q4: Degree variable is a categorical variable, but it is included in the regression as a continuous variable (as I understand from the results table). Authors should check which variables are continuous and categorical .

Re: In this paper, we control the CEO's educational background in the regression model. Higher education can improve CEOs' overall quality and professionalism, making them more decisive and proactive in enterprise management. In the opinion of the Board of Directors, such CEOs tend to be more capable, dare to take risks, and make correct decisions, which may be conducive to improving firm performance. In addition, when choosing CEOs, enterprises also trust CEOs with a better educational background more and give them more rights. Therefore, CEOs with better educational background have more say in improving firm performance. The selection of educational background in this paper is the highest degree of CEO. The highest education refers to the highest degree (such as junior college degree, bachelor's degree, master's degree, or doctor's degree) that the CEO has obtained up to the time the sample is used. The higher the degree, the higher the degree score. According to the research of Fan et al., CEO education level is a score ranging between 1 and 5: 1 denotes a degree below junior college; 2 denotes a junior college degree; 3 denotes a bachelor's degree; 4 denotes a master's degree; 5 denotes a doctoral degree.

*Fan JP, Wong TJ, Zhang T. Politically connected CEOs, corporate governance, and Post-IPO performance of China's newly partially privatized firms. J Financ Econ. 2007;84(2):330-57.*

Q5: It is good to include Tobin’s Q variable as another proxy for the firm performance. However, there is no discussion about it. How is it calculated? Is it only an alternative to ROA and ROE (for robustness purposes) or another performance proxy such as PEF? It would be useful to provide more discussion/explanations about it.

Re: In the robustness test, we use Tobin's q measure of firm performance in the robustness test as our dependent variable. Tobin's q ratio (or q as it is commonly known), defined as the capital market value of the firm divided by the replacement value of its assets incorporates a market measure of firm value which is forward-looking, risk-adjusted, and less susceptible to changes in accounting practices (64, 65).

*[64] Bharadwaj AS, Bharadwaj SG, Konsynski BR. Information technology effects on firm performance as measured by Tobin's q. Manage Sci. 1999;45(7):1008-24.*

*[65] Montgomery CA, Wernerfelt B. Diversification, Ricardian rents, and Tobin's q. The Rand journal of economics. 1988:623-32.*

Q6: Authors claim that they are using firm fixed effects regression in Section 4.3 based on Hausman test, but Table 3 suggests “Industry fixed effects”. I am not quite sure if authors use firm or industry fixed effects.

Re: This paper adopts the two-way fixed effects model. Yeardummy and Industrydummy represent the year dummy and industry dummy variables, respectively. Both of them are 0-1 dummy variables. uit is the model error term. i is the i - th firm. t is the t year. The above control and dummy variables are added to the model to control the influence of other factors further.

Q7: I am still not convinced about the endogeneity test. Authors provide some graphs (descriptive) about the relationship between CEO and top management teams changes, but it is not clear how it addresses endogeneity. What should readers understand from these graphs? Are there any other sources of endogeneity such as omitted variables, selection bias etc.? After explaining the Figure 2, authors state that “the model results still support Hypothesis 1 and 2”. I am not quite sure which model authors refer to and how it supports Hypothesis 1 and 2 .

Re: First, explanation of the graphs is as follows: A potential endogeneity problem with our findings is that listed companies have other executives besides the CEO. According to our statistics, the top management team changes almost every year. If the top management team is highly fluid, it is difficult to measure whether the CEO or other executives bring about an improvement in firm performance.

Therefore, we overcame the obstacles and selected the cleanest sample that was as undisturbed as possible. In these samples, it is guaranteed that the CEO was changed, but TMT remains relatively stable, avoiding the CEO appointment of TMT. This method can better describe the relationship between CEOs' functional experience and company performance.

Second, Instrumental Variable Analysis. Although the fixed effect model can solve the problem of missing variables to a certain extent. There may still be two-way causal problems. Without verification, we cannot rule out the possibility of a two-way causal relationship between firm performance and the CEO's functional experience. For example, due to the improvement of the firm's performance, it is possible to achieve a leading position in the whole industry or even more extensive cross-industry cooperation. As a result, CEOs have more opportunities for cross-border cooperation, thus enriching their functional experience. Of course, the above inference is only a possible guess. As for the possible two-way causal problem, this paper uses the instrumental variable method to test it further. An appropriate instrumental variable needs to meet two basic requirements: it is related to an endogenous variable (*func_rich*), and it is independent of other variables that cannot be observed but will affect the dependent variable (firm performance, *ROA*). This paper uses the average CEOs' functional richness (*func_mean*) in the same province and industry in the same year as an instrumental variable of the *func_rich*.

**Table 5 Regression results of instrumental variable**

|  | 1 | 2 |
| --- | --- | --- |
|  | **First step** | **Second step** |
|  | **func_rich** | **RO**A |
| func_rich | - | -0.0735*** |
|  | - | (-3.5437) |
| func_mean | 0.0802*** | - |
|  | -2.9601 | - |
| level_score | 0.0156*** | 0.0227*** |
|  | -2.9657 | -2.9946 |
| avg_emp | 0.2382*** | 0.1506** |
|  | -6.2684 | -2.1463 |
| gender | 0.0259** | 0.3501* |
|  | -2.4202 | -1.8561 |
| age | -0.0056** | -0.0651** |
|  | (-2.2341) | (-2.2304) |
| degree | 0.0659** | 0.0503** |
|  | -2.0637 | -2.0476 |
| sharend | 0.0135** | 0.0427** |
|  | -2.3407 | -2.0759 |
| dar | -0.2350*** | -0.3540* |
|  | (-7.5404) | (-1.8364) |
| size | 0.0240** | 0.2375** |
|  | -2.3541 | -2.2434 |
| nstaff | 0.1437* | 0.2413* |
|  | -1.7213 | -1.9834 |
| exe_num | 0.0357** | 0.3354* |
|  | -1.0254 | -1.9834 |
| found_time | 0.0127* | 0.4721** |
|  | -1.6923 | 2.0427 |
| _cons | -2.5473 | -1.9641 |
|  | (-0.0315) | (-0.3576) |
| Industry-year fixed effects | Yes | Yes |
| N | 27930 | 27930 |
| R-sq | 0.7653 | 0.6954 |
| adj. R-sq | 0.7754 | 0.6642 |

Table 5 reports the regression results using the instrumental variable. Column (1) of Table 5 reports the regression results of the first stage of instrumental variables. It can be seen from column (1) of Table 5 that *func_mean* regression coefficient is significantly positive at 1%, meeting the correlation conditions. At the same time, Cragg Donald Wald F statistic result shows 1736.93, and Kleibergen Paap Wald rk F statistic result shows 1006.22, both of which reject the hypothesis of weak instrumental variables. Column (2) of Table 5 reports the regression results of the second stage of instrumental variables. It can be seen from column (2) of Table 5 that *func_rich* regression coefficient is significantly positive at the level of 1%, which indicates that the research conclusion of this paper is still valid after using instrumental variables to solve potential endogenous problems.

Thank you for your patiently reviewing the manuscript. We are sincerely sorry for our carelessness. We have already corrected the description according to your valuable suggestion in the revised manuscript. We hope that you will be satisfied with the revision. Subsequently, we look forward that the revised manuscript being adaptable for the demands for open publication.

I want to re-submit this manuscript and hope it is acceptable for publication in the journal. If there are any questions about our paper, please do not hesitate to let us know. Thank you very much for your attention to our paper.

Best regards,

Xiaohong Huang

School of Logistics and Management Engineering

Yunnan University of Finance and Economics

Kunming, 650221, China

E-mail: huangxiaohong@163.sufe.edu.cn
